# Protection Induced by *Plasmodium falciparum* MSP1_42_ Is Strain-Specific, Antigen and Adjuvant Dependent, and Correlates with Antibody Responses

**DOI:** 10.1371/journal.pone.0002830

**Published:** 2008-07-30

**Authors:** Jeffrey A. Lyon, Evelina Angov, Michael P. Fay, JoAnn S. Sullivan, Autumn S. Girourd, Sally J. Robinson, Elke S. Bergmann-Leitner, Elizabeth H. Duncan, Christian A. Darko, William E. Collins, Carole A. Long, John W. Barnwell

**Affiliations:** 1 Division Malaria Vaccine Development, Walter Reed Army Institute of Research, Silver Spring, Maryland, United States of America; 2 Biostatistics Research Branch, National Institute of Allergy and Infectious Diseases, National Institutes of Health, Bethesda, Maryland, United States of America; 3 Division of Parasitic Diseases, Malaria Branch, National Center for Zoonotic, Vector-Borne and Enteric Disease, Centers for Disease Control, Atlanta, Georgia, United States of America; 4 Department of Malaria and Vector Research, National Institute of Allergy and Infectious Diseases, National Institutes of Health, Bethesda, Maryland, United States of America; University of California Los Angeles, United States of America

## Abstract

Vaccination with *Plasmodium falciparum* MSP1_42_/complete Freund's adjuvant (FA) followed by MSP1_42_/incomplete FA is the only known regimen that protects *Aotus nancymaae* monkeys against infection by erythrocytic stage malaria parasites. The role of adjuvant is not defined; however complete FA cannot be used in humans. In rodent models, immunity is strain-specific. We vaccinated *Aotus* monkeys with the FVO or 3D7 alleles of MSP1_42_ expressed in *Escherichia coli* or with the FVO allele expressed in baculovirus (bv) combined with complete and incomplete FA, Montanide ISA-720 (ISA-720) or AS02A. Challenge with FVO strain *P. falciparum* showed that suppression of cumulative day 11 parasitemia was strain-specific and could be induced by *E. coli* expressed MSP1_42_ in combination with FA or ISA-720 but not with AS02A. The coli42-FVO antigen induced a stronger protective effect than the bv42-FVO antigen, and FA induced a stronger protective effect than ISA-720. ELISA antibody (Ab) responses at day of challenge (DOC) were strain-specific and correlated inversely with c-day 11 parasitemia (r = −0.843). ELISA Ab levels at DOC meeting a titer of at least 115,000 ELISA Ab units identified the vaccinees not requiring treatment (noTx) with a true positive rate of 83.3% and false positive rate of 14.3 %. Correlation between functional growth inhibitory Ab levels (GIA) and cumulative day 11 parasitemia was weaker (r = −0.511), and was not as predictive for a response of noTx. The lowest false positive rate for GIA was 30% when requiring a true positive rate of 83.3%. These inhibition results along with those showing that antigen/FA combinations induced a stronger protective immunity than antigen/ISA-720 or antigen/AS02 combinations are consistent with protection as ascribed to MSP1-specific cytophilic antibodies. Development of an effective MSP1_42_ vaccine against erythrocytic stage *P. falciparum* infection will depend not only on antigen quality, but also upon the selection of an optimal adjuvant component.

## Introduction

*Plasmodium falciparum* is the leading cause of malaria morbidity and mortality. Each year more than 300 million cases of clinical malaria are reported, with deaths occurring primarily in children [1]. Because development of immunity to malaria is due, at least in part, to antibody (Ab) against erythrocytic stage parasites [2], a malaria vaccine should include components that induce such Ab. The major merozoite surface protein-1, MSP1, is a leading erythrocytic stage vaccine candidate [3], and is one of the most extensively studied of the malaria parasite antigens. It is secreted as a 195-kDa precursor that is membrane anchored via glycosylphosphatidylinositol [4] and is processed by proteases producing fragments of 83, 28–30, 38–45 and 42 kDa [5]–[7]. During merozoite invasion, the 42 kDa fragment (MSP1_42_) is further processed to produce a 33-kDa fragment (MSP1_33_), and a 19 kDa C-terminal fragment (MSP1_19_); the latter remains attached to the surface of the merozoite during invasion [8], [9].

MSP1_19_ is comprised of two tandem epidermal growth factor-like (EGF) domains [8] each containing three disulfide bridges [10], which force the assembly of several well-defined discontinuous epitopes [11]–[13]. Despite all that is known of MSP1 structure, little is known of its biological function, although there is evidence that MSP1 binds to erythrocytes and may have a role in erythrocyte invasion [14]–[16].

Evidence supporting the use of MSP1_42_ or its MSP1_19_ portion as a malaria vaccine is extensive. MSP1_19_-specific monoclonal Abs inhibit *P. falciparum* growth *in vitro* [17] and passively protect mice against infection with *P. yoelii*, [18], [19]. Immunization of *Aotus* monkeys with native *P. falciparum* MSP1, [20], baculovirus-expressed recombinant MSP1_42_ [21], [22], *E. coli* expressed MSP1_42_ [23], [24] or *Saccharomyces cerevisiae* recombinant MSP1_19_ [25], protect against homologous challenge. *E. coli-*expressed *P. yoelii* MSP1_19_ protects against a homologous challenge in rodent models [19], [26]. Anti-sera raised against recombinant MSP1_42_ inhibit *P. falciparum* growth *in vitro* [27]–[30], or inhibit secondary processing of MSP1_42_ [31]. MSP1_19_-specific antibodies from immune residents of malaria endemic areas also appear to play a predominant role in merozoite invasion inhibition [32]. Recently, however, the value of these *in vitro* assays has come into question because cytophilic MSP1-specific Ab appear to be more important for controlling infection than previously thought [33], [34].

In this current work we expand on previous work [24] to show the protective effect of vaccinating *Aotus* monkeys with three different recombinant p42 fragments of MSP1 varied according to both antigen (Ag) and adjuvant. Animals given 3D7 strain MSP1_42_, which is heterologous to the FVO challenge strain were not protected by vaccination, and animals given homologous strain FVO MSP1_42_ in combination with complete and incomplete Freund's adjuvant (FA) had greater chance of being protected than those given homologous Ag in combination with Montanide ISA-720. Animals given homologous Ag in combination with AS02A were not protected by vaccination. Protection correlated with ELISA-specific Ab measured with recombinant MSP1_42_ and its fragments, and correlated with growth inhibitory responses when measured against FVO but not the 3D7 strain of *P. falciparum.* In two separate experiments, animals given vaccine in combination with Montanide ISA-720 showed a significant capacity for inducing protection against infection, with most animals remaining untreated or treated for anemia. Our results may relate, in part, to these adjuvants' respective capacities for guiding the development of TH1 based immunity, and indicate that an effective malaria vaccine based on MSP1_42_ will depend not only on Ag quality but also adjuvant selection.

## Results

### Vaccination and Challenge

*Aotus* monkeys were immunized with various Ag/adjuvant combinations and challenged intravenously with 1×10^4^ FVO strain *P. falciparum* (Table 1, 2 and 3).

**Table 1:** Trial Design and Results for Adjuvant CFA/FA

Regimen	Ag	Monkey	Day 11	Tx Status	GIA		ELISA Ab Units		ELISA Ab Titer (*E.coli* expressed)			
					3D7 GIA	FVO GIA	bv42 (FVO)	bv42 (3D7)	EGF1	EGF2	p19	p42
1	Pvs25	2865	39,461	TxP	6.7%	−18.0%	5.35×10^1^	9.30×10^1^	n/d	n/d	n/d	n/d
		2869	125,361	TxP	13.1%	*	7.40×10^1^	7.90×10^1^	n/d	n/d	n/d	n/d
		2891	23,521	TxP	11.2%	−5.9%	3.95×10^1^	4.80×10^1^	n/d	n/d	n/d	n/d
		2909	99,231	TxP	3.1%	9.8%	3.48×10^1^	1.60×10^1^	n/d	n/d	n/d	n/d
		2916	18,761	TxP	16.5%	11.8%	1.04×10^2^	1.12×10^2^	n/d	n/d	n/d	n/d
2	coli42 (3D7)	2882	159,381	TxP	−2.4%	−2.7%	1.74×10^4^	2.21×10^4^	5.52×10^4^	3.50×10^3^	2.75×10^5^	3.54×10^5^
		2886	11,431	TxP	4.1%	0.1%	5.10×10^4^	6.42×10^4^	3.09×10^5^	3.01×10^4^	1.17×10^6^	1.53×10^6^
		2887	1,111	TxA	20.3%	21.7%	3.66×10^4^	4.07×10^4^	2.22×10^5^	2.42×10^4^	6.05×10^5^	6.95×10^5^
		2908	43,501	TxP	27.6%	18.5%	5.86×10^4^	9.72×10^4^	6.16×10^5^	6.24×10^4^	1.32×10^6^	2.11×10^6^
		2941	11,401	TxP	33.9%	48.1%	2.94×10^4^	5.80×10^4^	3.92×10^5^	2.19×10^4^	1.26×10^6^	1.22×10^6^
		2948	701	TxP	3.6%	−17.1%	2.12×10^4^	3.19×10^4^	1.68×10^5^	6.99×10^3^	3.53×10^5^	5.46×10^5^
3	coli42 (FVO)	2863	612	TxA	22.4%	45.6%	2.06×10^5^	1.18×10^5^	7.83×10^5^	6.32×10^4^	1.02×10^6^	4.36×10^6^
		2899	13	noTx	51.4%	47.3%	3.77×10^5^	3.09×10^5^	1.32×10^6^	2.95×10^5^	1.55×10^6^	8.34×10^6^
		2900	31	TxA	40.4%	34.2%	2.84×10^5^	2.79×10^5^	1.42×10^6^	6.96×10^4^	1.31×10^6^	5.37×10^6^
		2905	25	noTx	−1.9%	15.8%	1.15×10^5^	1.19×10^5^	1.12×10^6^	7.54×10^4^	1.44×10^6^	4.06×10^6^
		2929	34	noTx	63.7%	64.4%	2.68×10^5^	2.15×10^5^	1.23×10^6^	5.41×10^4^	1.18×10^6^	5.20×10^6^
		2931	13	noTx	27.5%	50.0%	2.38×10^5^	1.85×10^5^	1.28×10^6^	1.43×10^5^	1.47×10^6^	5.98×10^6^
7	bv42 (FVO)	2872	160	TxA	12.0%	27.9%	7.66×10^4^	6.50×10^4^	1.94×10^5^	2.03×10^4^	3.81×10^5^	1.53×10^6^
		2875	790	TxA	4.7%	24.4%	8.08×10^4^	5.71×10^4^	2.51×10^5^	8.96×10^3^	3.22×10^5^	1.44×10^6^
		2930	1,690	TxP	19.5%	48.3%	1.19×10^5^	1.04×10^5^	6.94×10^5^	5.81×10^3^	6.98×10^5^	2.62×10^6^
		2939	141	noTx	19.3%	28.3%	1.20×10^5^	9.71×10^4^	4.40×10^5^	1.12×10^4^	6.88×10^5^	2.77×10^6^
		2949	5,920	TxP	9.3%	50.5%	9.18×10^4^	6.83×10^4^	2.44×10^5^	1.22×10^4^	4.37×10^5^	2.31×10^6^

*Aotus* monkeys vaccinated with Ag in combination with FA. Groups of six animals were vaccinated. Day 11 is c-day 11 parasitemia. Tx is treatment outcome: noTx, no treatment required; TxA treatment for anemia; TxP, treatment for uncontrolled parasitemia. GIA is *in vitro* growth/invasion inhibition against FVO or 3D7 strain *P. falciparum.* ELISA Ab units were determined with 3D7 or FVO strain MSP1_42_ expressed in baculovirus. ELISA Ab titers were determined with 3D7 or FVO strain MSP1_42_, p19, EGF1 or EGF2 expressed in *E. coli*, and assays were run only using Ag homologous to the vaccine strain. Some results from regimens 1, 3 and 7 were reported previously [24]. n/d is not done. ^*^, no sample available.

**Table 2:** Trial Design and Results for Adjuvant AS02A.

Regimen	Ag	Monkey	Day 11	Tx Status	GIA		ELISA Ab Units		ELISA Ab Titer (*E.coli* expressed)			
					3D7 GIA	FVO GIA	bv42 (FVO)	bv42 (3D7)	EGF1	EGF2	p19	p42
4	Nothing	1784	368,931	TxP	−2.5%	12.1%	3.48×10^1^	2.30×10^1^	n/d	n/d	n/d	n/d
		1787	78,521	TxP	38.8%	13.4%	3.48×10^1^	2.00×10^1^	n/d	n/d	n/d	n/d
		2801	228,722	TxP	−10.7%	−3.4%	3.48×10^1^	1.40×10^1^	n/d	n/d	n/d	n/d
		2808	307,351	TxP	21.3%	*	3.48×10^1^	1.60×10^1^	n/d	n/d	n/d	n/d
		2885	314,931	TxP	8.6%	*	3.48×10^1^	9.00E+00	n/d	n/d	n/d	n/d
		2904	72,021	TxP	8.9%	16.2%	3.48×10^1^	8.00E+00	n/d	n/d	n/d	n/d
5	coli42 (3D7)	2789	86,581	TxP	11.9%	−9.9%	1.28×10^4^	1.17×10^4^	4.79×10^4^	2.78×10^2^	1.07×10^5^	1.02×10^5^
		2807	119,531	TxP	9.0%	−30.9%	1.12×10^4^	1.32×10^4^	3.09×10^4^	2.93×10^2^	1.08×10^5^	1.06×10^5^
		2890	143,611	TxP	16.1%	19.5%	1.11×10^3^	2.12×10^3^	6.29×10^3^	5.00×10^1^	1.25×10^4^	1.45×10^4^
		2902	70,491	TxP	13.5%	15.2%	7.94×10^3^	1.41×10^4^	2.83×10^4^	3.58×10^2^	1.02×10^5^	1.16×10^5^
		2921	26,771	TxP	39.6%	3.0%	4.04×10^4^	4.99×10^4^	2.46×10^5^	1.13×10^3^	4.81×10^5^	4.85×10^5^
		2943	12,731	TxP	2.0%	−10.8%	4.27×10^3^	6.70×10^3^	2.72×10^4^	5.00×10^1^	4.57×10^4^	4.93×10^4^
6	coli42 (FVO)	2884	141,451	TxP	−10.4%	−33.1%	5.29×10^3^	4.40×10^3^	2.89×10^4^	6.79×10^2^	4.59×10^4^	8.61×10^4^
		2901	10,351	TxP	12.9%	9.9%	4.67×10^3^	5.87×10^3^	2.81×10^4^	1.11×10^3^	4.59×10^4^	5.45×10^4^
		2903	24,541	TxP	*	*	4.43×10^3^	5.76×10^3^	4.13×10^4^	2.95×10^2^	5.51×10^4^	1.12×10^5^
		2914	128,271	TxP	36.2%	52.9%	6.95×10^3^	8.17×10^3^	6.07×10^4^	2.09×10^2^	9.24×10^4^	1.16×10^5^
		2926	111,721	TxP	−6.6%	5.5%	6.70×10^3^	8.24×10^3^	6.06×10^4^	2.54×10^2^	1.08×10^5^	1.67×10^5^

*Aotus* monkeys vaccinated antigens in combination with AS02A. Groups of six animals were vaccinated. Day 11 is c-day 11 parasitemia. Tx is treatment outcome: noTx, no treatment required; TxA treatment for anemia; TxP, treatment for uncontrolled parasitemia. GIA is *in vitro* growth/invasion inhibition against FVO or 3D7 strain *P. falciparum.* ELISA Ab units were determined with 3D7 or FVO strain MSP1_42_ expressed in baculovirus. ELISA Ab titers were determined with 3D7 or FVO strain MSP1_42_, p19, EGF1 or EGF2 expressed in *E. coli*, and assays were run only using Ag homologous to the vaccine strain.

n/d is not done. ^*^, no sample available.

**Table 3:** Trial Design and Results for Adjuvant Montanide ISA-720

Regimen	Ag	Monkey	Day 11	Tx Status	GIA		ELISA Ab Units		ELISA Ab Titer (*E.coli* expressed)			
					3D7 GIA	FVO GIA	bv42 (FVO)	bv42 (3D7)	EGF1	EGF2	p19	p42
8	coli42 (FVO)	1786	18,720	noTx	15.2%	35.9%	5.34×10^4^	4.39×10^4^	2.06×10^5^	2.43×10^4^	4.42×10^5^	1.06×10^6^
		2806	67,830	TxA	−13.1%	6.8%	3.64×10^4^	3.39×10^4^	1.98×10^5^	5.34×10^3^	3.74×10^5^	1.09×10^6^
		2876	600	TxP	−6.5%	9.9%	1.94×10^4^	1.73×10^4^	4.96×10^4^	6.09×10^3^	1.22×10^5^	3.01×10^5^
		2911	7,300	TxP	43.5%	7.9%	7.67×10^3^	9.29×10^3^	3.76×10^4^	2.27×10^3^	5.75×10^4^	1.84×10^5^
		2940	10,190	TxP	3.4%	41.2%	2.83×10^4^	2.55×10^4^	2.45×10^5^	1.97×10^4^	4.68×10^5^	7.28×10^5^
9	bv42 (FVO)	1788	10,040	TxA	0.5%	3.1%	6.34×10^4^	4.91×10^4^	4.51×10^5^	5.59×10^3^	3.81×10^5^	2.20×10^6^
		2792	118,810	TxP	41.1%	−33.6%	7.34×10^3^	6.69×10^3^	4.28×10^4^	2.45×10^2^	3.97×10^4^	1.24×10^5^
		2867	41,020	TxP	28.5%	27.7%	1.31×10^4^	1.15×10^4^	4.93×10^4^	5.17×10^2^	4.62×10^4^	5.13×10^5^
		2917	70,220	TxP	−2.4%	16.8%	1.44×10^4^	1.74×10^4^	4.13×10^4^	5.00×10^1^	4.67×10^4^	2.70×10^5^
		2920	121,370	TxP	38.3%	24.6%	2.59×10^3^	3.16×10^3^	1.34×10^4^	1.94×10^2^	1.15×10^4^	5.22×10^4^
		2942	24,150	TxP	8.3%	3.6%	7.25×10^3^	8.18×10^3^	5.04×10^4^	7.86×10^2^	4.44×10^4^	1.24×10^5^

*Aotus* monkeys vaccinated with one of three MSP1_42_ antigens in combination with ISA-720. Groups of six animals were vaccinated. Day 11 is c-day 11 parasitemia. Tx is treatment outcome: noTx, no treatment required; TxA treatment for anemia; TxP, treatment for uncontrolled parasitemia. GIA is *in vitro* growth/invasion inhibition against FVO or 3D7 strain *P. falciparum.* ELISA Ab units were determined with 3D7 or FVO strain MSP1_42_ expressed in baculovirus. ELISA Ab titers were determined with 3D7 or FVO strain MSP1_42_, p19, EGF1 or EGF2 expressed in *E. coli*, and assays were run only using Ag homologous to the vaccine strain.

### Affect of Vaccination on Treatment Outcome

Inspection of the results in Table 1, 2 and 3 shows that the different vaccination regimens induced responses having different effects on the possible treatment outcomes, which are defined as noTx (no treatment owing to self cure), TxA, (treatment for anemia), or TxP (treatment for uncontrolled parasitemia). This observation was supported by Fisher's exact test (p = 0.00046).

Only three of the vaccine regimens, regimen 3 (coli42-FVO/FA), regimen 7 (bv42-FVO/FA), and regimen 8 (coli42-FVO/ISA-720), induced any noTx responses. All three of these “protective” vaccines were comprised of Ag that is homologous to the challenge strain. Of these three regimens, only one had a matching regimen that matched on everything except for antigen strain (vaccine regimen 3, coli42-FVO, is matched with vaccine regimen 2, coli42-3D7). A Wilcoxon-Mann Whitney (WMW) test on treatment outcome was significantly better for the homologous antigen regimen (p = 0.0065). This suggests that the protective effect was strain-specific. Vaccines adjuvanted with AS02A did not induce a protective effect.

### Strain-specific MSP1_42_-specific ELISA Ab Responses

**Figure 1:**
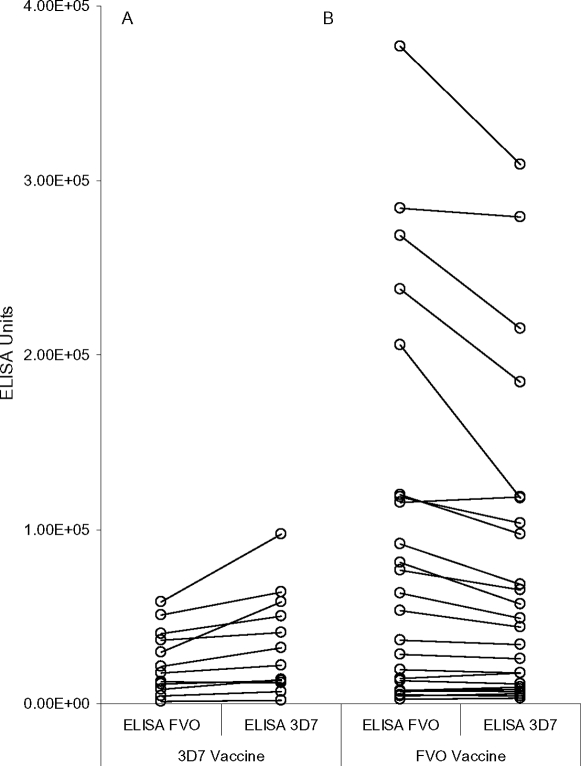
Individual value plots showing strain-specific Ab responses by ELISA for vaccine recipients. Responses are grouped by vaccine strain received and then by the Ag strain use for ELISA. Panel A includes all 3D7 strain vaccine recipients in the trial. Panel B includes all FVO strain vaccine recipients in the trial. Connect lines join each individual animal's ELISA Ab response measured with the FVO Ag to that measured with the 3D7 Ag. Both Ag were expressed in baculovirus.

Day of Challenge sera (DOC sera) were evaluated by ELISA against the 3D7 and FVO alleles of MSP1_42_ expressed in baculovirus to determine if the strain specific protective affect of vaccination was also apparent in the Ab responses. As shown in Fig. 1A and B, there is a general tendency for the Ab response against homologous Ag to exceed that measured against heterologous Ag (p<0.0001 by Wilcoxon signed rank test), especially for the samples containing higher Ab levels. These results can also be observed in Table 4, which, through a series of paired t-tests, also shows that the Ag/FA but not the Ag/AS02A or Ag/ISA-720 combinations induced significant strain-specific Ab responses.

**Table 4:** Group Wise Comparisons of Adjuvant Effect on Induction of Strain-specific Ab Responses

Adjuvant	Ag	Ag-specific ELISA Ab Units^1^		
		FVO (mean)	3D7 (mean)	paired t-test p
FA	coli42(3D7)	33,688	52,343	0.033
	coli42(FVO)	248,067	203,983	0.029
	bv42(FVO)	97,520	78,200	0.001
AS02A	coli42(3D7)	12,944	16,299	0.085
	coli42(FVO)	5,608	6,485	0.121
ISA-720	coli42(FVO)	29,026	25,963	0.162
	bv42(FVO)	18,008	15,990	0.462

1 ELISA Ab units were determined with 3D7 and FVO strain MSP1_42_ expressed in baculovirus.

### Models Evaluating the Affect of Adjuvant and Antigen on Response

We used a cumulative logit model to begin addressing the observation, from Table 1 and 3, that antigen and adjuvant affected treatment outcome. Because the study design was not balanced with respect to antigens and adjuvants, and because several vaccine regimens had all monkeys responding with the worst response (TxP), many of the simple models listed in Table 5 would not converge if all the data were included. Thus, the models were restricted to the twenty-two animals from vaccine regimens 3, 7, 8 and 9 (Table 1 and 3). These four vaccine regimens create a balanced 2 by 2 design, comparing two levels of antigen (bv42-FVO and coli42-FVO) with two levels of adjuvant (FA and ISA-720). Responses from these animals were treated as ordinal, with the best response being those that did not require treatment (noTx), the next better response being those treated for anemia (TxA), and the worst response being those treated for uncontrolled parasitemia (TxP). Model 3 (Table 5) had the lowest Akaike's Information Criterion (AIC), and the model says that the odds of making a response of noTx compared to either of TxA or TxP is 12.47 times greater for FA than for ISA-720 [95% confidence interval (1.96, 115.2)], regardless of the antigen (p = 0.0374). It further says that the odds of making a response of noTx compared to either of TxA or TxP is 6.71 times greater for coli42-FVO than for bv42-FVO [95% confidence interval (1.11, 53.5)], regardless of the adjuvant (p = 0.00649). Thus adjuvant and Ag both contributed significantly to the induction of responses that effected treatment outcome.

**Table 5:** Cumulative Logit Models used to Evaluate the Relationship between Treatment Outcome and Adjuvant and Antigen

Model 0	No effect of antigen and no effect of adjuvant
Model 1	Main effect from antigen but no effect from adjuvant
Model 2	No effect from antigen but main effect from adjuvant
Model 3:	Main Effects from both
Model 4:	Main effects plus an interaction term

**Figure 2:**
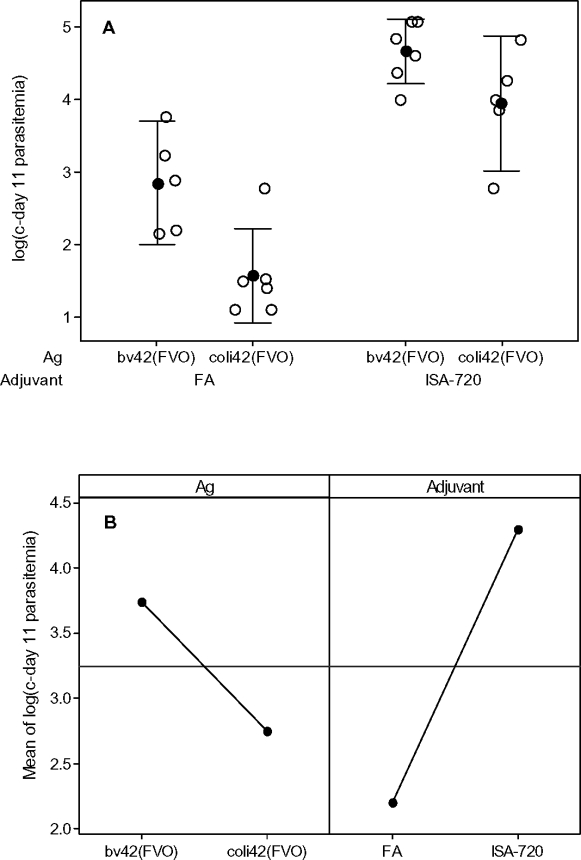
C-day 11 parasitemias for regimens 3, 7, 8, and 9 grouped by adjuvant and antigen. Open circles, individual responses; filled circles, means; bar is the 95% confidence interval for the mean. Panel A compares grouped log(c-day 11 parasitemia) levels. Panel B shows the estimated marginal means from the GLM used to analyze the responses.

Next we used a general linear model (GLM) to determine if antigen and adjuvant affected the log(c-day 11 parasitemia) among the same animals (regimens 3, 7, 8, and 9) as used in the cumulative logit model above. We tried the 5 models in Table 5 for this regression and also chose model 3 by AIC. The model showed a strong effect for adjuvant (ISA-720 had on average 1.9540 times higher log10(c-day 11 parasitemia) responses than FA, p<0.0001) and antigen (bv42-FVO had on average 1.3627 times higher log10(c-day 11 parasitemia) responses than coli42-FVO, p = 0.001). This model had a strong coefficient of determination (R^2^ (adjusted) = 0.7904). The responses evaluated with this model are shown graphically in (Fig. 2A). In Fig. 2B we show the marginal means from the model, where for example, the marginal mean for the bv42 antigen represents the modeled mean for that antigen averaged over both adjuvants. This model agrees qualitatively with the cumulative logit model, with both models showing that the beneficial effect of FA over ISA-720 is about twice as large as the beneficial effect of coli42-FVO over bv42-FVO.

**Figure 3:**
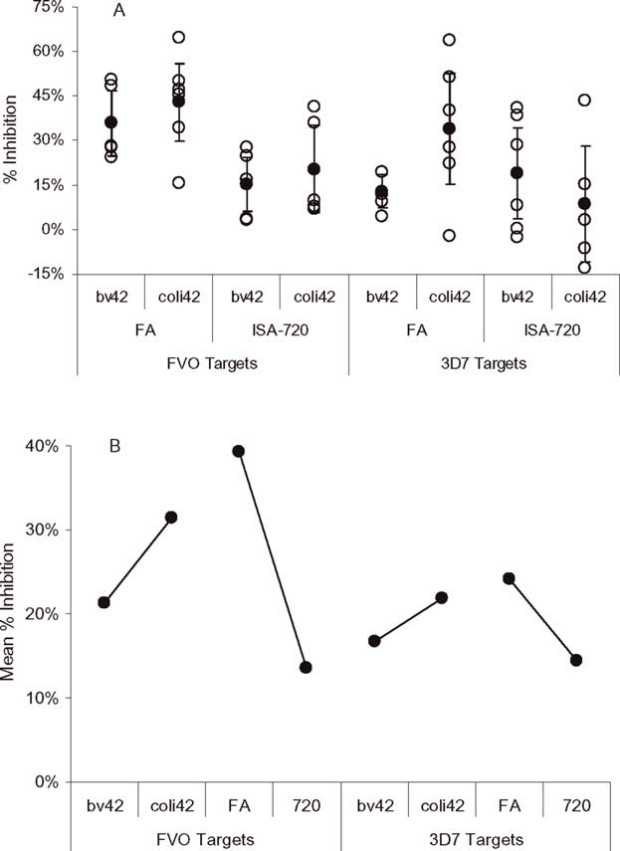
Growth/invasion inhibition for regimens 3, 7, 8, and 9 grouped by adjuvant and antigen. Open circles, individual responses; filled circles, means; bar is the 95% confidence interval for the mean. Panel A compares grouped %Inhibition levels. Panel B shows the estimated marginal means from the GLM used to analyze the responses.

**Figure 4:**
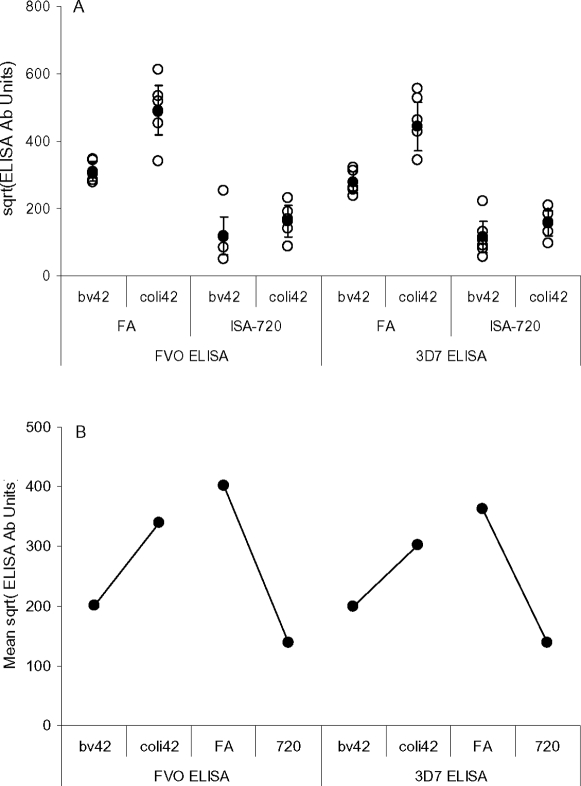
ELISA Ab responses for regimens 3, 7, 8, and 9 grouped by adjuvant and antigen. Capture Ags were expressed in baculovirus. Open circles, individual responses; filled circles, means; bar is the 95% confidence interval for the mean. Panel A compares grouped ELISA Ab unit levels. Panel B shows the estimated marginal means from the GLM used to analyze the responses.

We then used the same GLM (Model 3) to examine the affect of adjuvant and antigen on the induction of Ab responses that were inhibitory to FVO and 3D7 strain *P. falciparum* growth *in vitro* as well as those that were reactive in ELISA with various constructions of MSP1_42_ and its fragments. Growth inhibitory Ab responses did not require transformation for this analysis (Fig. 3A), while all of the ELISA Ab responses were transformed to the square root (Fig. 4A). In all cases variance testing could not rule out the assumptions that grouped responses were normally distributed and had the same variances.

When %Inhibition against FVO strain *P. falciparum* was the response term, the model showed a strong effect for adjuvant (FA had on average 2.9037 times higher %Inhibition responses than ISA-720, p<0.003) but affect of antigen was not significant (p = 0.185). This model had a weak coefficient of determination (R^2^ (adjusted) = 0.3819). The responses evaluated with this model are shown graphically in (Fig. 3A), with estimated marginal means shown in Fig. 3B. When %Inhibition against 3D7 strain *P. falciparum* was the response term, neither adjuvant nor antigen had a significant affect on the model (Fig. 3B).

Evaluation of the baculovirus-expressed FVO-ELISA Ab Units response in the GLM (Model 3) showed that both adjuvant and antigen had significant main effects on the model. FA had on average 2.8818 times higher FVO strain ELISA Ab responses than ISA-720 (p<0.001) and coli42-FVO had on average 1.6920 times higher FVO strain ELISA Ab responses than bv42-FVO, (p<0.001). The model had a strong coefficient of determination, (R^2^ (adjusted) = 0.9306). The responses evaluated with this model are shown graphically in Fig. 4A (left side); estimated marginal means are shown in Fig. 4B (left side). The same pattern of results and significances were obtained when this GLM was tested with 3D7 ELISA Ab responses, R^2^ = 0.771, Figs 4A and 4B (right side). For both models the affect of adjuvant was about 2 times greater than the affect of antigen.

### Correlations between c-day 11 parasitemia and serologic responses

**Figure 5:**
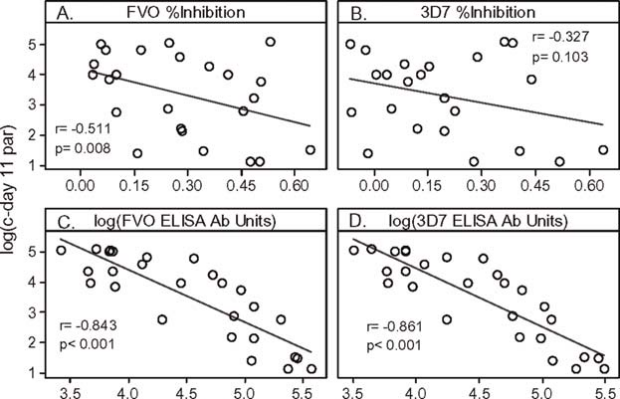
Correlation between log(c-day 11 parasitemia) and serologic response in animals vaccinated with FVO strain Ag. Animals were vaccinated with either *E. coli* or baculovirus expressed MSP1_42_ in combination with FA, ISA-720 or AS02A. Growth/Invasion inhibition is against FVO (Panel A) or 3D7 (Panel B) strain targets. ELISA was performed with recombinant FVO (Panel C) or 3D7 (Panel D) strain MSP1_42_ expressed in baculovirus. Correlations were determined with Pearson's test after testing data distributions for normality.

Pearson's tests on responses from the twenty-seven monkeys vaccinated with any FVO Ag in combination with any adjuvant (vaccine regimens 3, 6, 7, 8, 9 in Table 1 and 3) showed significant correlations between log(c-day 11 parasitemia) and the % Inhibition response against FVO strain but not 3D7 *P. falciparum* (Fig. 5A, r = −0.511, p = 0.008 and Fig. 5B, r = −0.327, p = 0.103, respectively). These tests also showed significant inverse correlations between c-day 11 parasitemia and ELISA Ab responses measured with both baculovirus expressed FVO strain and 3D7 strain MSP1_42_ (Fig. 5C, r = −0.843, p<0.001 and Fig. 5D, r = −0.861, p<.001, respectively)

We further investigated the possibility that each of the 4 pre-challenge covariates of Fig. 5 could be used for predicting an outcome of self-cure (noTx required) by evaluating Receiver Operation Characteristic (ROC) graphs (not shown). This grouping of animals contained six true positive results (self cure) and twenty-one true negative results (TxA or TxP). In Table 6, we show threshold values that give the smallest false positive rates given a true positive rate of 83.3% or greater. Neither GIA assay had good predictive value for predicting the outcome of noTx required because the false positive rate for the 3D7 assay and the FVO assay were 40% and 30%, respectively, when their threshold %Inhibition responses (15.4% and 28.3%, respectively) were set to detect 83.3% of true positives. The 3D7 and FVO MSP1_42_ ELISA assays were both predictive for the outcome of noTx, with both detecting a false positive rate of 14.3% when their threshold responses (97,100 ELISA units and 115,400 ELISA units, respectively) were set to detect 83.3% of true positives.

**Table 6:** Results from ROC graphs for Predicting noTx by Using GIA or ELISA with Ag Expressed in Baculovirus

Response	False Positive Rate	True Positive Rate	Threshold
% Inhibition −3D7 Targets	40%	83.3%	15.2% Inhibition
% Inhibition – FVO Targets	30%	83.3%	28.3% Inhibition
ELISA Ab Units −3D7 Strain	14.3%	83.3%	97,100 ELISA Units
ELISA Ab Units – FVO Strain	14.3%	83.3%	115,400 ELISA Units

Responses evaluated were from the twenty-seven animals in vaccine regimens 3, 6, 7, 8 and 9, vaccinated with any FVO Ag in combination with any adjuvant (Table 1, 2 and 3). Responses larger than the threshold value predict noTx outcome. %Inhibition is *in vitro* growth/invasion inhibition against FVO or 3D7 strain *P. falciparum.* ELISA Ab units were determined with 3D7 or FVO strain MSP1_42_ expressed in baculovirus.

### MSP1_42_ fragment-specific Ab Responses

In an attempt to improve the sensitivity and specificity of ELISA for detecting animals that would not require treatment we evaluated the ELISA Ab responses to *E. coli* expressed MSP1_42_ from the FVO strain of *P. falciparum* and its three fragments, MSP1_19_ (P19), EGF1, and EGF2. For the twenty-seven animals in vaccine regimens 3, 6, 7, 8 and 9, all of the ELISAs detected responses showed significant inverse correlations with c-day 11 parasitemia (data not shown, all r≤−0.808, all p≤0.001 ). Results for response levels giving a true positive rate of 83.3% or greater are shown in Table 7. Although all of the ELISA assays were predictive for the outcome of noTx, the assays based on measuring Ab to either EGF2 and MSP1_42_ were best. Both assays detected a false positive rate of 9.5% when their threshold ELISA Ab response levels (2.4×10^4^ and 2.7×10^6^, respectively) were set to detect 83.3% of true positives. Because of the small sample sizes, however, further work is needed to differentiate between the abilities of the different ELISA assays to predict outcome.

**Table 7:** Results from ROC graphs for Predicting Self-Cure by Using ELISA with FVO strain Ag expressed in *E. coli*

Response	False Positive Rate	True Positive Rate	Threshold for ELISA Ab Titer
ELISA Ab Titer – EGF1	19%	83.3%	4.0×10^5^
ELISA Ab Titer – EGF2	9.5%	83.3%	2.4×10^3^
ELISA Ab Titer – p19	14.3%	83.3%	6.5×10^5^
ELISA Ab Titer – p42	9.5%	83.3%	2.7×10^6^

Responses evaluated were from the twenty-seven animals in vaccine regimens 3, 6, 7, 8 and 9, vaccinated with any FVO Ag in combination with any adjuvant (Table 1, 2 and 3). ELISA Ab titers were determined with FVO strain MSP1_42_, p19, EGF1 or EGF2 expressed in *E. coli*, which are homologous to the vaccine strain.

### Protection Induced by coli42-FVO/ISA-720

**Figure 6:**
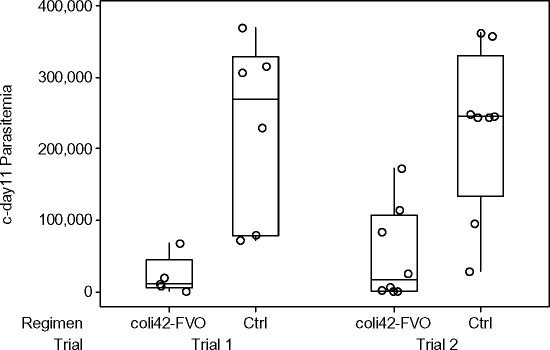
Responses in animals vaccinated with coli42-FVO/ISA-720 or control. Shaded Open circles indicate individual responses, the boxed area shows defines the median and the second and third quartile responses, and whiskers define the first and fourth quartile responses.

We were most interested in determining if the protective effect induced by the coli42-FVO/ISA-720 regimen (Table 3) was significant because of the possibility that this combination would be acceptable for human use (Fig. 6, Trial 1). WMW tests showed that c-day 11 parasitemia levels were significantly lower among animals receiving coli42-FVO/ISA-720 (median 1.0×10^4^ parasites/μl) than among AS02A controls (median 2.7×10^5^ parasites/μl, p = 0.004).

A second trial (Fig. 6, Trial 2) was designed to confirm our observation of the protective effect of coli42-FVO/ISA-720 by including a proper ISA-720 control and by using more animals (N = 8). In the first trial in the coli42-FVO/ISA-720 group, one of five animals did not require treatment, one was treated for anemia, and three were treated for uncontrolled parasitemia (Table 3). In the second trial two of seven animals did not require treatment, three were treated for anemia, and two were treated for uncontrolled parasitemia. The eighth animal, which appeared to be controlling its infection, died prematurely during handling (data not shown). WMW tests showed that c-day 11 parasitemia levels were significantly lower among animals receiving coli42-FVO/ISA-720 (median 1.6×10^4^ parasites/μl) than among animals receiving ISA-720 adjuvant alone (median 2.5×10^5^ parasites/μl, p = 0.0054). No differences in parasitemia were observed between the control groups (p = 1.0000) or the vaccine groups (p = 0.9417) of the two trials. These results confirm that coli42-FVO/ISA-720 induces some protective immunity in *Aotus* monkeys.

## Discussion

Our primary objectives for this study were to determine if vaccination with *E. coli* expressed MSP1_42_-FVO or −3D7 or baculovirus expressed MSP1_42_-FVO in combination with FA, or the human use adjuvants AS02A or ISA-720 induced a protective immune response against FVO strain *P. falciparum* challenge. Secondly we sought to identify Ab dependent correlates of protection.

The overarching results of the trial were that the protective effects of vaccination are strain-specific and depended on the combination of adjuvant and Ag used for vaccination. Both FVO strain antigens used in this study protected against FVO challenge but 3D7 strain antigen did not, and the antigens also induced antibodies having a significant strain-specific component as measured by ELISA. One of the protective combinations was coli42-FVO/ISA-720, which is compatible with human use. Although observed previously in rodent models of malaria [35]–[37], this is the first time that a *P. falciparum* challenge model has shown that a *P. falciparum* erythrocytic stage vaccine can induce a significant protective effect when combined with an adjuvant considered suitable for human use.

Statistical models evaluating the requirement to treat (cumulative logit model) or the suppression of c-day 11 parasitemia (GLM) showed that both adjuvant (FA superior to ISA-720) and antigen (coli42-FVO superior to bv42-FVO) affected the induction of protective effects against erythrocytic stage FVO strain *P. falciparum*. The GLM for ELISA Ab response (capture antigens expressed in baculovirus) showed that for both homologous and heterologous MSP1_42_ Ag, the level of Ab induction was both adjuvant and Ag dependant. ROC calculations prepared with results from vaccine recipients given any FVO strain MSP1_42_ Ag/Adjuvant combination showed that p42-specific and EGF2-specific ELISA Ab levels (capture antigens expressed in *E. coli*) had the best predictive value for identifying animals with an outcome of noTx, with both detecting a false positive rate of 9% when their threshold responses (ELISA Ab titer = 2.7×10^6^ and 2.4×10^3^, respectively) were set to detect 83.3% of true positives. The small improvement obtained by using the MSP1_42_ Ag expressed in *E. coli* over that obtained with MSP1_42_ Ag expressed in baculovirus may be due to either chance or to simple protein glycosylation by baculovirus. Study design did not allow us to formally examine the contributions of AS02A adjuvant or coli42-3D7 Ag to the induction of protective responses; however, inspection of the results in Tables 1 and 2 indicates that neither of these factors participated in this process.

In apposition to the argument that immunity was strain specific, is that protection depends only on immunogenicity. ANOVA (data not shown) indicates that, when tested by ELISA with all permutations of capture Ag strain, the immunogenicity of coli42-3D7/FA was significantly lower than that of coli42-FVO/FA, but the immunogenicities of the protective coli42-FVO/ISA-720 combination and the non-protective coli42-3D7/FA combination were not different. This suggests that coli42-3D7/FA should have been able to induce a measurable protective effect if immunogenicity is the controlling factor rather than the induction of strain dependent immunity.

We used a GLM to show that the induction of growth inhibitory Ab was adjuvant dependant (FA superior to ISA-720) but not antigen dependent. Similarly to what has been observed previously, c-day 11 parasitemia correlated inversely with growth inhibition against FVO strain *P. falciparum* [28]. Similar but less extreme and non-significant inverse correlations were seen for 3D7 strain *P. falciparum.* The difference in these correlations might be attributable to MSP1_42_ related strain-dependant differences in the mechanisms of invasion/growth inhibition [30]. ROC graphs showed that GIA assays were not as useful for predicting an outcome of noTx as ELISA assays.

The affect of adjuvant on the induction of protection is complex and requires further study. ELISA reactive Ab correlated well with the induction of protective immunity as shown previously [28]. However it was the Ag/FA combinations that had the greatest affect on the induction of these responses. Five of the six animals that self cured their infections received FA as the adjuvant, and these five animals all had Ab levels exceeding the threshold values for predicting noTx from the ROC graphs. Furthermore, Ag/FA combinations induced more Ab reactive with homologous Ag than heterologous Ag, but Ag/AS02A or Ag/ISA-720 combinations did not show this affect.

FA may have supported the induction of a strong TH1 based immune response and appears to have a higher capacity for inducing such responses than Montanide ISA-720 which in turn seems to be more potent in this regard than AS02A [38]. This property of FA further supports the importance of the Fc region in Ab-mediated immunity against erythrocytic stage malaria infection [34]. Inflammatory responses induce by FA, such as induction of IFN-gamma and TGF-beta, play a critical role in Ig class switching and thus in the promotion of cytophilic Ab responses [39]. Recently, a chimeric mouse model for human immunity against *P. falciparum* was used to show that the Ab Fc region plays an important role in Ab mediated protection against erythrocytic stage malaria parasite infection [34]. Furthermore, this protection was unrelated to the induction of invasion inhibitory Ab; Fc fragment mediated processes are not measured in most GIAs [34].

Adjuvant selection will be a critical component of MSP1_42_ based vaccine development for humans. There is a proposal that IFA be used for vaccinating residents of malaria endemic areas against *P. falciparum* [40]. However, if the induction of TH1 based immunity is, indeed, an important component of the induction of protective immunity, it does not appear that IFA offers an effective strategy [41], [42] unless it is supplemented with stimulants such as CpG [42]. Other, perhaps more suitable, adjuvant possibilities include Montanide ISA-720 supplemented with CpG [43] or AS01B [44].

## Materials and Methods

### Recombinant Expression Plasmids

Plasmids for expression of GST/EGF-like domain 1 and GST/EGF-like domain 2 from the 3D7 and FVO strains of *P. falciparum* MSP1 and p19 from the FVO strain were constructed previously [45]. The plasmid for expression of GST/ MSP1_19_ (3D7) was prepared by cloning the appropriate PCR-synthesized fragment into the BamHI (5') and NotI (3') sites of pGEX-6p. The insert was sequenced to verify reading frame and primary structure.

**Recombinant protein expression and purification** MSP1_42_-FVO and -3D7 were expressed in baculovirus (bv) or *E. coli* (coli) and purified as described previously [22], [24], [31], [46]. GST fusion proteins corresponding to the p19, EGF1, and EGF2 fragments of MSP1_42_ were expressed in *E. coli* and purified as described previously [24].

### Vaccination and Challenge of *Aotus* Monkeys

Groups of six animals were immunized with coli42-FVO, coli42-3D7, bv42-FVO, or the *P. vivax* sexual stage protein Pvs25 given with Freund's Complete and Incomplete adjuvant (FA), Montanide ISA-720 (Seppic, ISA-720), or AS02A (GSK) (Table 1, 2 and 3). Pre-immune sera were collected 2 weeks (Time -2) prior to the first immunization. Animals given Ag/AS02A or Ag/ISA-720 combinations were vaccinated at weeks 0, 4, 12, and 24. Those given Ag/FA combinations were vaccinated at week 12 with Ag/complete FA and at week 19 with Ag/incomplete FA. Vaccinations were with 50 μg of Ag. All animals were challenged intravenously at week 26 with 1×10^4^ FVO strain *P. falciparum* taken from a donor monkey. Generally, the severity of the response induced by Freund's adjuvants in *Aotus* monkeys limits the number of immunizations to no more than three. It was our intention to vaccinate the monkeys given Ag/FA combinations three times at four week intervals beginning at week twelve of the study, and then challenge two weeks after the last immunization. However, unexpectedly severe responses after priming forced us to delay the second immunization by three weeks and ultimately to eliminate the third immunization. Nevertheless, these animals were challenged at the same time as the one given Ag mixed with the other adjuvants.

Beginning 3 days after the challenge, parasite density was quantified from Giemsa-stained thick blood films; when parasite counts exceeded 80,000/μL of blood, parasite densities were quantified from thin blood smears. Blood smears were prepared daily and evaluated for 56 days after the challenge. Monkeys that developed high-density parasitemia (TxP, 200,000 parasites/μL of blood) were treated with mefloquine (Roche Laboratories, Nutley, NJ) and quinine (Marion Merrel Dow, Inc., Kansas City, KS). Animals that developed anemia (TxA) were cured with drugs and treated by iron supplementation and transfusion of whole blood after the hematocrit fell below 20%. Animals that resolved parasitemia without treatment are denoted noTx. Sera for the ELISA and GIA analyses were collected on the day of challenge (DOC).

### ELISA Serology

ELISAs for detecting MSP1_42_-specific Ab responses have been described previously [47] as have the ELISAs for detecting MSP1_42_ domain-specific Ab responses (to p19, EGF1 and EGF2) with *E. coli* expressed Ag [24].

### Parasite Invasion/Growth Inhibition

3D7 and FVO *P. falciparum* parasites were maintained asynchronously in media consisting of RPMI 1640 (Invitrogen) with 25 mM HEPES, 7.5% w/v NaHCO_3_ and 10% A^+^ pooled human serum in A^+^ E at 2–4% hct [48]. Parasites were synchronized with a 40/70/90 percent Percoll gradient two days prior to assay and the growth inhibitory activity of the sera measured as described previously [49] after modification to reduce sample volumes [50].

### Statistical Methods

Fisher's exact test was used to determine if treatment outcomes (noTx, no treatment owing to self cure; TxA, treatment for anemia; TxP, treatment for uncontrolled parasitemia) varied according to vaccine regimen. A cumulative logit model was used to evaluate the influence of adjuvant and antigen on the ordered treatment outcome (ordering: noTx, TxA, TxP). The five models that were compared are listed in Table 5. The best model was picked by AIC [51]. An exact Wilcoxon-Mann-Whitney (WMW) test was used when treatment outcomes were compared between only two vaccine regimens.

Induction of strain specific Ab responses as measured by ELISA was evaluated by using the Wilcoxon signed rank test. Cumulative day 11 parasitemia (c-day 11 parasitemia) was calculated by summing daily parasitemia from the day of challenge (DOC) until the day the first animal in the study was treated for any reason (day 11). It was used as the continuous variable for evaluating the correlation between serological responses and parasite responses. General linear models (GLM) were used to evaluate the influence of adjuvant and antigen on the induction of responses (c-day 11 parasitemia, growth inhibition, ELISA Ab level). For these analyses c-day 11 parasitemia was transformed by log_10_. For the GLM used to evaluate the relationship between the affect of antigen and adjuvant on the ELISA Ab response level, log transformations were not normally distributed. Instead, Box-Cox analysis showed that the ideal transformation was “square root”. Growth inhibition responses were not transformed for testing in the GLM. All correlations were evaluated with Pearson's test after testing responses for normality with the Kolgomorov-Smirnov test. The calculations for the cumulative logit model were done using the MASS package version 7.2–29 in R version 2.4.0. ROC calculations were also prepared in R but using the ROCR package [52]. All other calculations were done using MiniTab Statistical Software, Release 14.1. All p-values are two-sided.
